# 

**Published:** 1895-11-16

**Authors:** 


					THE HOSPITAL. ABSOLUTELY PURE, therefore BEST. November 16th, 1895.
CADBURY'S ^ WW ^ CADBURY'S
O COCOA JJfI COCOA
Of absolute purity and freedom from alka'i." _ ]??) t?J cHj " The Typical Cocoa of English Manufacture-
?Braithuaitt. Absolutely Pure."?The Analyst.
WHICH HOSPI TAL
No. 477. Vol. XIX.
Satukday,
Nov. 16th, 1895.
WITH EXTRA NURSING SUPPLEMENT. twopence.
^ ? , _ . __ ? . Ver Annum, 10s. 6d? post rree.
[Registered at the General Post Office as a Newspaper for Monthly Parts, 6d.; post free, 8d.
transmission in the United Kingdom and Abroad.] . nittoper Annum, 8s.6d? post frea.

				

